# Maternal sleep practices and stillbirth: Findings from an international case‐control study

**DOI:** 10.1111/birt.12416

**Published:** 2019-01-18

**Authors:** Louise M. O’Brien, Jane Warland, Tomasina Stacey, Alexander E. P. Heazell, Edwin A. Mitchell, the STARS Consortium, JH Collins, JL Huberty, HJ Kliman, JA McGregor, M Parast, M Peesay, LJ Wimmer

**Affiliations:** ^1^ Division of Sleep Medicine, Department of Neurology, and Department of Obstetrics and Gynecology Michigan Medicine Ann Arbor Michigan; ^2^ Mothers, Babies and Families Research Group, School of Nursing and Midwifery University of South Australia Adelaide South Australia Australia; ^3^ School of Healthcare University of Leeds Leeds UK; ^4^ St. Mary’s Hospital, Manchester Academic Health Science Centre Manchester University NHS Foundation Trust Manchester UK; ^5^ Maternal and Fetal Health Research Centre, School of Medical Sciences, Faculty of Biology, Medicine and Health University of Manchester Manchester UK; ^6^ Department of Paediatrics, Child and Youth Health University of Auckland Auckland New Zealand

**Keywords:** maternal sleep, sleep duration, stillbirth

## Abstract

**Background:**

Late stillbirth, which occurs ≥28 weeks’ gestation, affects 1.3‐8.8 per 1000 births in high‐income countries. Of concern, most occur in women without established risk factors. Identification of potentially modifiable risk factors that relate to maternal behaviors remains a priority in stillbirth prevention research. This study aimed to investigate, in an international cohort, whether maternal sleep practices are related to late stillbirth.

**Methods:**

An Internet‐based case‐control study of women who had a stillbirth ≥28 weeks’ gestation within 30 days before completing the survey (n = 153) and women with an ongoing third‐trimester pregnancy or who had delivered a live born child within 30 days (n = 480). Bivariate and multivariate logistic regressions were used to determine unadjusted and adjusted odds ratios (OR and aOR, respectively) with 95% confidence intervals (95% CIs) for stillbirth.

**Results:**

Sleeping >9 hours per night in the previous month was associated with stillbirth (aOR 1.75 [95% CI 1.10‐2.79]), as was waking on the right side (2.27 [1.31‐3.92]). Nonrestless sleep in the last month was also found to be associated with stillbirth (1.73 [1.03‐2.99]), with good sleep quality in the last month approaching significance (1.64 [0.98‐2.75]). On the last night of pregnancy, not waking more than one time was associated with stillbirth (2.03 [1.24‐3.34]). No relationship was found with going to sleep position during pregnancy, although very few women reported settling in the supine position (2.4%).

**Conclusions:**

Long periods of undisturbed sleep are associated with late stillbirth. Physiological studies of how the neuroendocrine and autonomic system pathways are regulated during sleep in the context of late pregnancy are warranted.

## 1 INTRODUCTION

The mean annual rate of reduction of late stillbirth, at 28 weeks’ gestation or later, has been approximately 2.3% in high‐income countries and remains between 1.3 and 8.8 per 1000.^1^ If all high‐income countries were able to achieve stillbirth rates that were the same as the best six performing countries, almost 20 000 third‐trimester stillbirths could have been avoided in 2015 alone.^1^ Established risk factors for stillbirth include advanced maternal age, maternal obesity, smoking, and maternal medical and obstetric conditions such as diabetes and preeclampsia.^2^ However, given that most of these factors cannot be modified during pregnancy, there is a cogent need to identify modifiable risk factors, such as maternal behaviors and lifestyle.^3^

In 2011, the Auckland Stillbirth Study first reported that maternal sleep position was a significant risk factor for late stillbirth.^4^ This case‐control study demonstrated that women who reported settling to sleep in the supine position on the last night of pregnancy were significantly more likely to experience stillbirth, even after accounting for other factors (aOR 2.54 [95% CI 1.04‐6.18]). Indeed, compared with women who went to sleep on their left side, those who went to sleep in any other position had double the risk for stillbirth (2.03 [1.24‐3.29]). This finding has now been observed in three further studies using similar methodologies with similar effect sizes.^5^, ^6^, ^7^ These studies have also noted differences in maternal sleep duration and number of awakenings between stillbirths and live births.

Following the publication of the Auckland Stillbirth Study,^4^ an international group of researchers and clinicians—the Study of Trends and Associated Risks for Stillbirth Consortium—partnered with the Star Legacy Foundation and other stillbirth and parental support groups to conduct a web‐based survey of women who had experienced a stillbirth using a nested case‐control design within an uncontrolled cohort.^8^, ^9^ The overall goal was to investigate potentially modifiable risk factors, including maternal sleep practices, for late stillbirth in a large, international population. To understand maternal behaviors and symptoms during pregnancy, we sought to obtain information directly from women themselves including items that would not otherwise be documented in medical records. Our primary hypothesis for the case‐control arm of the study was that supine sleep and long sleep duration would increase the risk for late stillbirth.

## 2 METHODS

**Figure 1:**
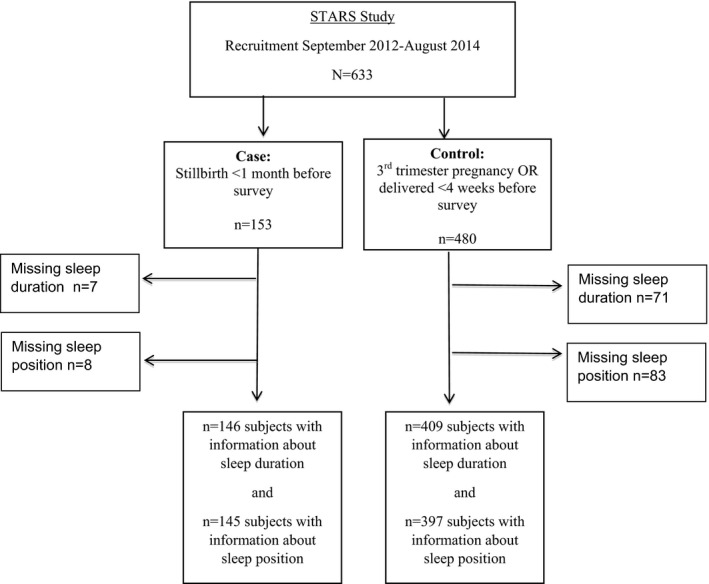
Participant flow diagram

An anonymous online survey—Study of Trends and Risk Factors for Stillbirth—was developed during the first Stillbirth Summit in Minneapolis in 2011 by an international consortium of clinicians and academics, together with the Star Legacy Foundation and other stillbirth and parental support groups.^8^ The participant flow diagram for this case‐control study is presented in Figure 1. Briefly, the survey included questions related to established risk factors for stillbirth, including smoking, maternally perceived changes in fetal movements,^9^ and maternal health conditions, and novel questions relating to emerging risk factors, such as maternal sleep. Several questions about maternal sleep practices before pregnancy, in the last month of pregnancy, and on the last night were included in the survey. For cases, the last night referred to the last night before realization of the stillbirth. To allow similar gestational ages in cases and controls, control women included those who were currently in their third trimester and those who had recently delivered. For controls that were still pregnant, the last month and night referred to the previous month and night before completion of the survey, respectively. For controls that had recently delivered their baby, these questions referred to the last month and last night of their pregnancy.

In addition to sleep and wake position, women were asked about their typical nighttime sleep duration, daytime nap duration, number of awakenings, number of times out of bed to use the bathroom, restless sleep, sleep quality, and whether medications were used to aid sleep. Habitual snoring was defined as snoring at least three nights/week, and the Epworth Sleepiness Scale^10^ was used to determine excessive daytime sleepiness. The Brief Restless Legs Scale,^11^ a 4‐item questionnaire, was used to identify the presence of restless leg syndrome. Questions were included with open‐ended responses, categorical responses (ie, yes/no/don’t know), Likert scales, drop‐down menus with a single response, or checkboxes that allowed multiple responses. Free text boxes were also provided to allow women to provide any additional information.

The questionnaire is available as Appendices S1 and S2 to the manuscript. In reporting this study, guidelines from strengthening the Reporting of Observational Studies in Epidemiology^12^ group were followed.

### 2.1 Participants

Women were invited to participate between September 2012 and August 2014 by web‐based advertising and social media by means of the Star Legacy Foundation and word of mouth. Cases were women at least 18 years old, fluent in reading and writing English, who had delivered a singleton stillborn baby at least 28 weeks’ gestation within 1 month before completing the survey. Controls were women at least 18 years old who were either still pregnant (28 weeks or more) or had delivered a living baby within the month before survey completion. Women less than 18 years old, those with a multifetal gestation, a fetus with known congenital anomaly, and those who were not fluent in reading or writing English were excluded. This study was approved by the Institutional Review Board of the University of Michigan. Participants accessed the survey after reading the purpose of the study and clicking on the “I agree” button, thereby providing consent. They were also provided with contact information for a stillbirth support group (First Candle) if they considered completion of the survey to be upsetting.

### 2.2 Sample size

Sample size for maternal sleep practices was calculated based on the anticipated exposure of supine sleep. With an exposure frequency of 20%, a sample of 144 cases and controls would be required to detect a difference in odds ratio of at least 3.0 between women with late stillbirths compared with healthy controls, assuming a power of 80%, *P* = 0.05.

### 2.3 Data analysis

Data were cleaned by two authors (JW and LMO) before analysis. Women found not to fit the a priori inclusion criteria above were excluded. Categorical variables were reported as counts and proportions, whereas continuous variables were presented as mean (standard deviation) or median (interquartile range). All statistical analysis was performed with SPSS (version 24; IBM, Armonk, NY, USA) using cross‐tabulations, with chi‐squared tests and logistic regressions to find unadjusted and adjusted odds ratios (OR and aOR, respectively) with 95% confidence intervals (95% CIs). The level of statistical significance was set at *P* < 0.05. A multivariate logistic regression model was developed to include demographic variables reported to be associated with increased risk of stillbirth, based on prior knowledge and previous literature (maternal age, educational level, smoking, body mass index [BMI], parity). Country of respondent (United States vs non‐United States) and ethnicity of respondent (Caucasian vs non‐Caucasian) were also added to the model as they were significantly different between cases and controls. Customized birthweight centile was calculated using GROW software.^13^ The customized birthweight centile was not included in the final multivariate model as birthweight data were missing for half of controls. A sensitivity analysis was carried out to compare whether incorporating the customized birthweight centile altered the findings.

## 3 RESULTS

During the study period, 153 cases and 480 controls completed the survey. The median duration of time since stillbirth was 13 days (range 1‐29 days). Participant demographics are shown in Table 1. The median gestation at the time of the stillbirth was 37 weeks (range 28‐41 weeks), and 52% of stillborn babies were male.

**Table 1:** Demographic information of an international group of women who had experienced a stillbirth of at least 28 weeks’ gestation compared with control women between 2012 and 2014

	Stillbirth (n = 153) n (%), Mean ± SD or Median [IQR]	Control (n = 480) n (%), Mean ± SD or Median [IQR]
Maternal age (years)	31 ± 5.4	30 ± 4.8
Maternal race		
Caucasian	122 (79.7)^**^	430 (90.2)
Non‐Caucasian	31 (20.3)	47 (9.8)
Median maternal BMI (kg/m^2^)	27 [23‐32]	25 [23‐31]
Median gestational age (weeks)	37 [34‐39]	37 [32‐39]
Median number of prior pregnancies	1 [0‐6]^***^	1 [0‐10]
Median number of prior live births	0 [0‐4]^**^	1 [0‐10]
Hypertension	6 (3.9)	35 (7.3)
Diabetes	18 (11.8)^*^	28 (5.8)
Smoking during pregnancy	20 (13.1)	41 (8.5)
Alcohol during pregnancy	33 (21.6)	134 (27.9)
Over‐the‐counter medication at least weekly	22 (14.4)^*^	111 (23.0)
Prescription medications at least weekly	30 (19.6)	127 (26.5)
Country^b^		
United States	123 (80.4)^**^	325 (67.7)
Other	30 (19.6)	148 (30.8)
Highest level of education		
Graduate education	35 (22.9)	129 (26.9)
College‐level education	81 (52.9)	242 (50.4)
High school or lower	37 (24.2)	106 (22.1)
Activity level during pregnancy		
Inactive	17 (11.1)	49 (10.3)
Lightly active	96 (62.7)	302 (62.9)
Moderately active	28 (18.3)	97 (20.2)
Active	7 (4.6)	18 (3.7)
Bedrest	4 (2.6)	10 (2.1)
Customized birthweight centile^a^		
<10th centile	36 (23.5)^***^	13 (2.7)
10‐49.9th centile	59 (38.5)^***^	72 (15.0)
50‐89.9th centile	35 (22.9)	117 (24.4)
≥90th centile	20 (13.1)	33 (6.9)
Missing	3 (2.0)	245 (51.0)

IQR, interquartile range; SD, standard deviation.

Several variables have a small number of missing values not shown, except birth centile, which is shown in the table.

a Customized birthweight centile is the birthweight‐for‐gestational‐age percentile that accounts for the influence of maternal characteristics on fetal growth.

b Other countries included in the survey are as follows: the United Kingdom (n = 95), Canada (n = 44), Australia (n = 21), New Zealand (n = 2), Germany (n = 2), Greece (n = 2), India (n = 2), Philippines (n = 2), South Africa (n = 2), Finland (n = 1), Italy (n = 1), Sweden (n = 1), Switzerland (n = 1), Israel (n = 1), and Bahrain (n = 1).

*P < 0.05; **P < 0.01; ***P < 0.001.

Compared with controls, women in the stillbirth group were less likely to be Caucasian (90.2% vs 79.7%, *P* < 0.0002). Indeed, Caucasian race was protective for stillbirth (OR 0.41 [95% CI 0.25‐0.68]). However, nulliparity was associated with stillbirth (1.80 [1.22‐2.64]). In the control women for whom birthweight was available, compared with babies born between 50th and 89.9th customized centile, those with birthweight <10th customized centile, birthweight between 10th and 49.9th customized centile, and birthweight >90th customized centile were all associated with stillbirth (9.26 [4.43‐19.37], 2.74 [1.64‐4.57], and 2.03 [1.04‐3.97], respectively).

### 3.1 Sleep variables

Before pregnancy, no differences were found in self‐reported sleep practices between cases and controls (see Table S1). Nocturnal sleep duration was significantly longer in cases compared with controls in the last month of pregnancy, as was total 24‐hour sleep duration over the same period (Table 2). This was driven by the nocturnal sleep duration as nap durations were not different between groups. Women in the stillbirth group were at significantly higher odds (aOR 1.75 [95% CI 1.10‐2.79]) of having long sleep duration (≥9 hours) over the previous month (Table 3) after adjustment for other variables although no relationship was found with sleep duration on the last night and stillbirth (Table 4). Those who had a stillbirth were more likely to report that they did not wake up or woke up only once on the last night (aOR 2.03 [95% CI 1.24‐3.34]). Epworth Sleepiness Scale scores (Table 2) and clinical levels of daytime sleepiness increased from prepregnancy (see Table S1) to the last month of pregnancy (Table 3) although remained similar between groups.

**Table 2:** Mean sleep variables before and during pregnancy in an international sample of women with and without a stillbirth, 2012‐2014

Variable	Stillbirth (n = 153) Mean ± SD	Controls (n = 480) Mean ± SD
Sleep duration		
Sleep duration before pregnancy (hours)	7.7 ± 0.9	7.6 ± 1.0
Sleep duration last month (hours)	7.9 ± 1.3**	7.5 ± 1.5
Sleep duration last night (hours)	6.4 ± 2.1	6.7 ± 2.1
Nap duration		
Nap duration before pregnancy (hours)	0.9 ± 1.0	1.0 ± 0.9
Nap duration last month (hours)	1.6 ± 0.8	1.6 ± 0.9
Total 24‐h sleep duration		
Total sleep in 24 h before pregnancy (hours)	8.7 ± 1.4	8.6 ± 1.4
Total sleep in 24 h last month (hours)	9.5 ± 1.5*	9.1 ± 1.8
Epworth Sleepiness Scale (ESS)		
Total ESS before pregnancy	3.9 ± 3.0	3.9 ± 3.1
Total ESS last month	7.5 ± 4.9	7.0 ± 4.5

* *P* < 0.05;

** *P* < 0.01.

**Table 3:** Odds of stillbirth by sleep variable in the last month of pregnancy in an international sample of women with and without a stillbirth, 2012‐2014

Variable	Stillbirth (n = 153) n (%)	Controls (n = 480) n (%)	Unadjusted OR (95% CI)	Adjusted OR (95% CI)
Sleep duration last month				
≤6 h	15 (9.8)	47 (9.8)	1.05 (0.56‐1.97)	1.11 (0.57‐2.16)
6.5‐8.5 h	86 (56.2)	283 (59.0)	Reference	Reference
≥9 h	45 (29.4)	79 (16.5)	1.87 (1.21‐2.91)	1.75 (1.10‐2.79)
Awakenings last month				
≤1 awakening	23 (15.0)	61 (12.7)	1.06 (0.63‐1.79)	1.13 (0.65‐1.97)
≥2 awakenings	121 (79.1)	341 (71.0)	Reference	Reference
Get up last month				
≤1 time up	47 (30.7)	129 (26.9)	1.00 (0.67‐1.51)	1.16 (0.75‐1.79)
≥2 time up	98 (64.1)	270 (56.3)	Reference	Reference
Restless last month				
None or little restless	52 (34.0)	97 (20.2)	1.71 (1.02‐2.88)	1.73 (1.03‐2.99)
Average restless	32 (20.9)	102 (21.3)	Reference	Reference
More than average or very restless	62 (40.5)	201 (41.9)	0.98 (0.60‐1.60)	0.91 (0.54‐1.53)
Fall asleep position last month				
Left	81 (52.9)	209 (43.5)	Reference	Reference
Supine	1 (0.7)	8 (1.7)	0.32 (0.04‐2.62)	0.37 (0.04‐3.12)
Right	37 (24.2)	95 (19.8)	1.00 (0.64‐1.59)	1.14 (0.70‐1.85)
Propped	5 (3.2)	14 (2.9)	0.92 (0.32‐2.64)	1.20 (0.39‐3.68)
Prone	0 (0)	5 (1.0)	NA	NA
Variable	21 (13.7)	66 (13.8)	0.82 (0.47‐1.43)	0.87 (0.48‐1.55)
Wake up position last month				
Left	40 (26.1)	128 (26.7)	Reference	Reference
Supine	11 (7.2)	42 (8.8)	0.84 (0.40‐1.78)	1.10 (0.50‐2.43)
Right	47 (30.7)	82 (17.1)	1.83 (1.11‐3.04)	2.27 (1.31‐3.92)
Propped	2 (1.3)	7 (1.5)	0.91 (0.18‐4.58)	0.98 (0.18‐5.30)
Prone	1 (0.7)	5 (1.0)	0.64 (0.07‐5.64)	0.51 (0.05‐4.87)
Variable	36 (23.5)	111 (23.1)	1.04 (0.62‐1.74)	1.16 (0.67‐2.00)
Naps last month				
Never/rare	38 (24.8)	125 (26.0)	Reference	Reference
Occasional	41 (26.8)	108 (22.5)	1.25 (0.75‐2.08)	1.32 (0.77‐2.77)
Often/almost always	67 (43.8)	168 (35.0)	1.31 (0.83‐2.08)	1.40 (0.86‐2.29)
Excessive daytime sleepiness last month				
No	97 (63.4)	281 (58.5)	Reference	Reference
Yes	42 (27.5)	107 (22.3)	1.14 (0.74‐ 1.74)	1.00 (0.64‐1.58)
Habitual snoring last month				
None/rare	68 (44.4)	196 (40.8)	Reference	Reference
Occasionally	34 (22.2)	95 (19.8)	1.03 (0.64‐1.67)	1.00 (0.59‐1.68)
Often/almost always	31 (20.3)	89 (18.5)	1.00 (0.61‐1.64)	0.95 (0.55‐1.66)
Restless leg syndrome last month				
No	92 (60.1)	255 (53.1)	Reference	Reference
Yes	44 (28.8)	128 (26.7)	0.95 (0.63‐1.45)	1.11 (0.71‐1.72)
Sleep quality last month				
Good/very good	48 (31.4)	89 (18.5)	1.69 (1.04‐2.75)	1.64 (0.98‐2.75)
Average	50 (32.7)	134 (27.9)	Reference	Reference
Poor/very poor	47 (30.7)	193 (40.2)	0.65 (0.41‐1.03)	0.65 (0.40‐1.06)
Medications to aid sleep last month				
No	129 (84.3)	345 (71.9)	Reference	Reference
Occasionally	8 (5.2)	25 (5.2)	0.86 (0.38‐ 1.95)	0.61 (0.25‐ 1.49)
Frequently	5 (3.3)	24 (5.0)	0.56 (0.21‐ 1.49)	0.64 (0.23‐ 1.45)

All models were adjusted for maternal age, educational level, smoking, body mass index (BMI), parity, country of respondent (United States vs non‐United States), and ethnicity. Sleep duration was reported in half‐hour increments.

**Table 4:** Odds of stillbirth by sleep variable in the last night of pregnancy in an international sample of women with and without a stillbirth, 2012‐2014

Variable	Stillbirth (n = 153) n (%)	Controls (n = 480) n (%)	Unadjusted OR (95% CI)	Adjusted OR (95% CI)
Sleep duration last night				
≤6 h	67 (43.8)	153 (31.9)	1.37 (0.91‐2.06)	1.31 (0.84‐2.03)
6.5‐8.5 h	59 (38.6)	184 (38.3)	Reference	Reference
≥9 h	16 (10.5)	54 (11.3)	0.92 (0.49‐1.74)	0.75 (0.39‐1.46)
Awakenings last night				
≤1 awakening	41 (26.8)	63 (13.1)	2.16 (1.37‐3.41)	2.03 (1.24‐3.34)
≥2 awakenings	94 (61.4)	312 (65.0)	Reference	Reference
Get up last night				
≤1 time up	60 (39.2)	132 (27.5)	1.43 (0.96‐2.13)	1.47 (0.96‐2.27)
≥2 time up	76 (49.7)	239 (49.8)	Reference	Reference
Restless last night				
None or little restless	47 (30.7)	90 (18.8)	1.24 (0.73‐2.10)	1.16 (0.66‐2.04)
Average restless	35 (22.9)	83 (17.3)	Reference	Reference
More than average or very restless	62 (40.5)	214 (44.6)	0.69 (0.42‐1.12)	0.66 (0.40‐1.11)
Fall asleep position last night				
Left	75 (49.0)	193 (40.2)	Reference	Reference
Supine	4 (2.6)	11 (2.3)	0.94 (0.29‐3.03)	1.05 (0.32‐3.50)
Right	45 (29.4)	111 (23.1)	1.04 (0.67‐1.62)	1.11 (0.70‐1.77)
Propped up	4 (2.6)	15 (3.1)	0.69 (0.22‐2.13)	0.71 (0.22‐2.30)
Prone	0 (0)	4 (0.8)	NA	NA
Variable	10 (6.5)	39 (8.1)	0.66 (0.31‐1.39)	0.75 (0.34‐1.64)
Wake up position last night				
Left	46 (30.1)	147 (30.6)	Reference	Reference
Supine	13 (8.5)	39 (8.1)	1.07 (0.52‐2.17)	1.25 (0.58‐2.73)
Right	44 (28.8)	97 (20.2)	1.45 (0.89‐2.36)	1.54 (0.91‐2.61)
Propped up	2 (1.3)	11 (2.3)	0.58 (0.12‐2.72)	0.56 (0.12‐2.70)
Prone	2 (1.3)	3 (0.6)	2.13 (0.35‐13.14)	1.87 (0.27‐12.83)
Variable	11 (7.2)	46 (9.6)	0.76 (0.37‐1.60)	0.88 (0.41‐1.90)
Restless leg syndrome last night				
No	100 (65.4)	292 (60.8)	Reference	Reference
Yes	20 (13.1)	73 (15.2)	0.80 (0.46‐1.38)	0.92 (0.51‐1.65)
Sleep quality last night				
Good/very good	37 (24.2)	67 (14.0)	1.53 (0.89‐2.60)	1.40 (0.79‐2.47)
Average	42 (27.5)	116 (24.2)	Reference	Reference
Poor/very poor	65 (40.5)	202 (42.1)	0.89 (0.57‐1.39)	0.89 (0.55‐1.43)
Medications to aid sleep last night				
No	138 (90.2)	367 (76.5)	Reference	Reference
Yes	6 (3.9)	24 (5.0)	0.67 (0.27‐1.66)	0.65 (0.26‐1.67)

All models were adjusted for maternal age, educational level, smoking, body mass index (BMI), parity, country of respondent (United States vs non‐United States), and ethnicity. Sleep duration was reported in half‐hour increments.

No relationship was found between reported position that women fell asleep in and stillbirth either in the last month or on the last night. However, the number of supine sleepers in this study was universally low (n = 1 for cases and n = 8 controls in the last month and n = 4 in the cases and n = 11 in the controls for the last night). Before pregnancy, the frequency of falling asleep supine was similar between groups (10.5% of cases and 12.7% of controls) but fell across both groups to 0.7% and 1.7% in the last month and 2.6% and 2.3% on the last night in cases and controls, respectively. This unexpected low frequency prevents full analysis. Wake up position was similar between groups before pregnancy, but cases were more likely to report waking up on their right side in the last month of pregnancy compared with controls (aOR 2.27 [95% CI 1.31‐3.92]) but not the last night (Tables 3 and 4). Position on going to sleep and position on waking up were highly correlated before pregnancy, in the last month, and on the last night (Pearson correlation coefficient *r* = 0.77, *r* = 0.81, and *r* = 0.83, respectively, *P* < 0.0001).

Both habitual snoring (snoring often or every night) and restless leg syndrome were similar in cases and controls at all time points and increased during pregnancy compared with before pregnancy (Table S1 and Tables 3 and 4). Compared with self‐report of an average amount of restless sleep, not having restless sleep was more likely in the stillbirth group in the last month, even after accounting for other risk factors (aOR 1.73 [95% CI 1.03‐2.99]).

Before pregnancy, sleep quality was similar between groups (Table S1), but poor sleep quality became more frequent during pregnancy (Tables 3 and 4). In the last month of pregnancy, the odds of good/very good sleep quality was higher in the stillbirth group (OR 1.69 [95% CI 1.04‐2.75]) although the difference was not significant after adjustment (Table 3). A minority of women reported use of medications during the last month and on the last night to help them sleep, but no relationship was found with stillbirth (Tables 3 and 4). A sensitivity analysis using customized birthweight centile as a covariate in the adjusted models described above did not appreciably change any of the findings.

To investigate whether there was a differential response to the survey from control women who had already delivered when they completed the survey compared with those who were still pregnant, a sensitivity analysis was conducted using only those women who were still pregnant compared with cases. This analysis did not alter the findings, and thus, all controls were retained. Moreover, a sensitivity analysis restricted to women in the United States only did not change any of the results.

No interactions were found between long sleep duration and fetal compromise, such as those born <10th centile or those exposed to maternal smoking. Furthermore, no interactions were found between long sleep duration and maternal perception of changes in fetal movement.

Women who had experienced a stillbirth were asked about their perception of the time that they believed their baby died. The time periods were “Morning, 6 am‐12 noon,” “Afternoon, 12 noon‐6 pm,” “Evening, 6 pm‐10 pm,” and “During the night, 10 pm‐6 am.” A total of n = 33 women (21.6%) were not sure and n = 8 women (5.2%) did not provide a response. However, out of the n = 112 women who provided a time period, n = 83 (74%) believed that their baby died during the night (Table 5).

**Table 5:** Perceived time of death in an international sample of women with a stillbirth, 2012‐2014

Time period	N = 153 n (%)
Morning (6 am‐12 noon)	10 (6.5)
Afternoon (12 noon‐6 pm)	11 (7.2)
Evening (6 pm‐10 pm)	8 (5.2)
During the night (10 pm‐6 am)	83 (54.2)
Not sure	33 (21.6)
Missing	8 (5.2)

## 4 DISCUSSION

This international study provides further evidence that maternal sleep practices are associated with a late stillbirth. In agreement with other studies, it suggests that long periods of undisturbed sleep, such as long sleep duration and not waking more than once, independent of other risk factors are associated with late fetal demise.

No evidence was found that maternal supine sleep position was a risk of stillbirth. Since the initial publication,^4^ one small cohort study from Ghana^14^ and several case‐control studies from Australia,^5^ New Zealand,^6^ and the United Kingdom^7^ have all demonstrated an association between supine sleep position and late stillbirth with odds ratios between 2.3 and 8.0. Although one of the goals of the current study was to investigate the role of supine sleep position, its reported frequency was much lower (2.3%) than the anticipated 20%. Thus, the study was underpowered to detect a difference at a low frequency of exposure.

As maternal sleep practices were of interest in the development of this case‐control study, a large number of questions about sleep were included. This allowed for exploration of changes in sleep across pregnancy since women were queried about sleep practices before pregnancy, in the last month, and on the last night. As expected, sleep disturbance increased during pregnancy. There is a large literature demonstrating that maternal sleep disturbance, such as sleep‐disordered breathing, short sleep duration, and poor sleep quality are common during pregnancy and have strong associations with poor outcomes such as gestational hypertension and preeclampsia,^15^, ^16^, ^17^, ^18^ gestational diabetes,^16^, ^19^, ^20^ fetal growth restriction,^21^, ^22^, ^23^ and preterm birth.^17^, ^21^, ^24^, ^25^ Importantly, the latter outcomes are known risk factors for stillbirth.^26^ Since nocturnal events may influence pregnancy outcomes, possibly by induction of inflammatory and oxidative stress responses, endothelial damage, and metabolic derangement,^27^, ^28^, ^29^ the link between maternal sleep and stillbirth warrants urgent investigation. Of note, consistent with other studies,^5^, ^8^ a large proportion of women (74%) perceived that their baby died during the night.

Our findings that long periods of undisturbed sleep were more frequent in the stillbirth group support other data. Indeed, the Auckland study^4^ reported higher odds of long sleep duration, one or fewer times getting up to use the bathroom, and regular daytime naps in the stillbirth group compared with controls, although short sleepers were also more common in the stillbirth group. This latter study was remarkably similar to the recent Midlands and North of England Stillbirth Study,^7^ which also reported frequent daytime napping, one or fewer awakenings to use the bathroom, and both long and short sleep as being more common in the stillbirth group. Short sleep—but not long sleep—was more common in the stillbirth group in the New Zealand multicenter stillbirth case‐control study,^6^ which also found a higher odds for not getting up to go to the toilet. Similar to the current study, McCowan et al^6^ did not find a relationship between daytime naps and stillbirth. Our novel findings of good sleep quality and lack of restless sleep being more likely in the stillbirth group compared with controls further add to this growing literature that suggests a role for undisturbed maternal sleep in stillbirth.

Prolonged sleep duration in older populations has been hypothesized to compensate for poor sleep quality^30^ although in the current study mothers who had a stillbirth were more likely to have good sleep quality. Long sleep durations have also been associated with a sedentary lifestyle, low levels of physical activity, socioeconomic status, and anxiety/depression.^30^, ^31^, ^32^ Nonetheless, in the context of stillbirth, the current study found no differences between cases and controls for reported levels of activity nor for educational attainment, but no measures of anxiety/depression were obtained.

Interestingly, otherwise healthy long sleepers have been reported to have elevated serum melatonin and cortisol, longer durations with low body temperature, and systematic differences in arousal.^33^ A strong relationship exists between arousal and an increase in sympathetic activity and thus blood pressure.^34^ Blood pressure decreases during sleep, with a gradual decrease during each stage of slow‐wave sleep irrespective of posture^35^ with the lowest pressure reached during deep sleep.^36^ During rapid eye movement sleep, blood pressure is transiently increased although not to awake levels. Indeed, the time spent in specific sleep states and the distribution of the sleep states across the night is known to affect blood pressure.^37^ Since arousals and awakenings from sleep cause surges in sympathetic activity with resultant increases in blood pressure, it is plausible that awakenings and periods of getting out of bed (such as to use the toilet) in pregnant women could serve to maintain blood pressure and prevent long periods of relative low pressures. This has particular relevance since maternal hypotension has been reported to be associated with fetal growth restriction, premature birth, and stillbirth.^38^, ^39^, ^40^ In a case‐control study designed to specifically investigate daytime maternal blood pressure in stillbirths compared with live births, Warland et al^41^ found that the stillbirth group were more likely to have borderline diastolic blood pressure (60‐70 mm Hg). Furthermore, the odds of stillbirth in women with at least three mean arterial pressure values ≤83 mm Hg was almost double that of controls (aOR 1.78 [95% CI 1.06‐2.99]). Moreover, long sleep duration may prolong inferior vena cava compression and lack of compression relief from not waking up could potentiate this effect.^42^

A strength of the current study is that it is the first to include an international group of women and the only one to include women from the United States; indeed, the majority of women received their care in the United States. The United States‐based Star Legacy Foundation hosted the online survey, and one possible explanation for the unexpectedly low prevalence of reported supine sleep potentially could have been because the results of the Auckland Stillbirth Study,^4^ the first to report an association between supine sleep and late stillbirth, were available on the website. Nonetheless, despite the international nature of this study we were unable to investigate the effect of race since the vast majority of women were of Caucasian background and insufficient women of other races were represented. Since non‐Hispanic black women are at 2‐3 times higher risk of stillbirth than are non‐Hispanic white women,^43^ there is an opportunity for future studies to explore sleep practices in the context of racial and ethnic background. Although part of the higher risk of fetal mortality for non‐Hispanic black women relates to their higher risk of preterm delivery, most of the disparity in fetal mortality remains unexplained.^44^

This study is not without limitation. Inherent in the design, it was only available to women who had Internet access. However, given that the Internet is widely available across the majority of household income brackets and that approximately one quarter of women had either a high school education or lower, the design is unlikely to limit generalizability. Although this was an international study, it is acknowledged that the majority of women were based in the United States. An additional potential limitation is recall bias, particularly with regard to sleep behaviors. However, care was taken to minimize recall bias by limiting the study to women who had delivered a stillborn baby within the previous month, when events can be recalled.^45^ Similarly, most controls were either still pregnant—to have a comparable range of gestational ages—or had delivered within the previous month. Although this approach cannot eliminate recall bias, use of similar time frames is unlikely to bias one group more than the other. Moreover, these data were based on subjective measures. Although objective measures of sleep such as polysomnography or actigraphy would allow a detailed understanding of nocturnal body position, it is extremely difficult to use such measures when stillbirth is the outcome of interest, since many thousands of women would be required for adequate power; this is cost‐prohibitive and logistically challenging. In addition, since the study included a large number of questions that were not related to sleep, it is unlikely that report of sleep practices would have been biased. Importantly, sleep practices before pregnancy were the same between groups.

In summary, long periods of undisturbed maternal sleep are associated with late stillbirth. Although no role for supine sleep was found—possibly because of very low numbers of women with reported supine sleep—findings of long sleep duration and few awakenings are consistent with other published data. Maternal sleep offers a modifiable risk factor for stillbirth. Physiological studies of how the neuroendocrine and autonomic system pathways are regulated during sleep in the context of late pregnancy are warranted.
