# Supplementary material for: Maternal sleep practices and stillbirth: Findings from an international case‐control study
Source: Birth. 2019 Jan 18;46(2):344–54. doi: 10.1111/birt.12416 (PMC7379524; doi:10.1111/birt.12416)
Supplement: Supplementary file 1 [file BIRT-46-344-s001.docx]

**Self report of sleep variables before pregnancy in an international sample of women with and without a stillbirth, 2012-2014:**

**Variable Stillbirth (n=153) Controls (n=480)**

**n (%) n (%)**

**Sleep Duration before pregnancy:**

≤6 hours 10 (6.5%) 38 (2.5%)

6.5-8.5 hours 120 (74.4%) 324 (67.5%)

≥9 hours 16 (10.5%) 49 (10.2%)

**Awakenings before pregnancy:**

≤1 awakening 121 (79.1%) 331 (69.0%)

≥2 awakenings 23 (15.0%) 78 (16.3%)

**Get up before pregnancy:**

≤1 time up 135 (88.2%) 363 (75.6%)

≥2 time up 10 (6.5%) 41 (8.5%)

**Restless before pregnancy:**

None or little restless 101 (66.0%) 262 (54.6%)

Average restless 27 (17.6%) 85 (17.7%)

More than average or very restless 18 (11.8%) 63 (13.1%)

**Fall Asleep Position before pregnancy:**

Left 22 (14.4%) 45 (9.4%)

Supine 16 (10.5%) 61 (12.7%)

Right 15 (9.8%) 53 (11.0%)

Propped 0 (0%) 1 (0.2%)

Prone 55 (35.9%) 156 (32.5%)

Variable 33 (21.6%) 90 (18.8%)

**Wake Up Position before pregnancy:**

Left 12 (7.8%) 37 (7.7%)

Supine 24 (15.7%) 76 (15.8%)

Right 16 (10.5%) 32 (6.7%)

Propped 0 (0%) 0 (0%)

Prone 23 (15.0%) 101 (21.0%)

Variable 61 (39.9%) 136 (28.3%)

**Naps before pregnancy:**

Never/Rare 121 (79.1%) 304 (63.3%)

Occasional 20 (13.1%) 81 (16.9%)

Often/Almost Always 4 (2.6%) 22 (4.6%)

**Excessive Daytime Sleepiness before pregnancy:**

No 136 (88.9%) 377 (78.5%)

Yes 6 (3.9%) 27 (5.6%)

**Habitual Snoring before pregnancy:**

None/Rare 98 (64.1%) 290 (60.4%)

Occasionally 35 (22.9%) 80 (16.7%)

Often/Almost Always 9 (5.9%) 31 (6.5%)

**Restless Leg Syndrome before pregnancy:**

No 122 (79.7%) 349 (72.7%)

Yes 16 (10.5%) 40 (8.3%)

**Sleep Quality before pregnancy:**

Good/Very Good 101 (66.0%) 295 (61.5%)

Average 37 (24.2%) 85 (17.7%)

Poor/Very Poor 7 (4.6%) 30 (6.3%)

**Medications to Aid Sleep before pregnancy:**

No 115 (84.3%) 318 (66.3%)

Occasionally 18 (5.2%) 66 (13.8%)

Frequently 9 (3.3%) 21 (4.4%)

Sleep duration was reported in half-hour increments.
