# Supplementary material for: Maternal sleep practices and stillbirth: Findings from an international case‐control study
Source: Birth. 2019 Jan 18;46(2):344–54. doi: 10.1111/birt.12416 (PMC7379524; doi:10.1111/birt.12416)
Supplement: Supplementary file 3 [file BIRT-46-344-s003.pdf]

The STARS Study

Thank you for your interest in participating in the STARS Study. Your participation will be valuable in helping to learn more about the causes of stillbirth.

These first few questions are to gather some general information about you at the time of this pregnancy.

What is today's date?

	MM		DD		YYYY
Please enter today's date.		/		/	

What is your date of birth?

	MM		DD		YYYY
Please enter date you were born.		/		/	

How tall are you? (Please indicate feet/inches or meters)

	Feet	Inches	Meters
Please enter your height in either feet/inches or centimeters			

How would you describe your race/ethnicity?

- ◯ Caucasian
- ◯ African American/Black
- ◯ Alaska Native
- ◯ Native American
- ◯ American or Canadian First Nations
- ◯ Asian
- ◯ Latino/Hispanic
- ◯ Pacific Islander
- ◯ Middle Eastern
- ◯ Australian Aboriginal
- ◯ Mixed race

Other (please specify)

In what country were you living during this pregnancy?

What is your highest level of education completed?

- ○ Some high school
- ○ High School Diploma
- ○ Associates Degree
- ○ Technical/Trade School Degree
- ○ Bachelor's Degree
- ○ Master's Degree
- ○ Doctorate Degree
- ○ Post-doctoral education

Other (please specify)

[ ]

How would you describe your employment status during this pregnancy?

- ○ Unemployed
- ○ Stay at home Mom.
- ○ Worked from home part time.
- ○ Worked from home full time.
- ○ Worked outside the home part time.
- ○ Worked outside the home full time.
- ○ Other (please specify)

[ ]

If you worked outside of the home during this pregnancy, was it shift work?

- ○ No
- ○ Yes, day shift
- ○ Yes, evening shift
- ○ Yes, night shift
- ○ Yes, rotating shifts
- ○ I do not work outside of the home.

The STARS Study

If you live in the United States, were you covered by private health insurance for this pregnancy?

◯ Yes

◯ No

If you live outside please describe any financial coverage for this pregnancy.

What is your combined household annual income in US dollars before taxes?

- ◯ Less than $25,000
- ◯ $25,000 - $50.000
- ◯ $50,000 - $100,000
- ◯ $100,000 - $200,000
- ◯ More than $200,000
- ◯ Prefer not to answer

For those outside the US - please enter your salary in your local currency.

[ ]

Are you in a stable relationship with this baby's father?

- ◯ yes
- ◯ no

Comment

[ ]

How old is the father of your child? (If unknown, do not answer)

How would you describe the father of this baby's race/ethnicity?

- ◯ Caucasian
- ◯ African American/Black
- ◯ Alaskan Native or American Indian
- ◯ Asian
- ◯ Latino/Hispanic
- ◯ Pacific Islander
- ◯ Middle Eastern
- ◯ Australian Aboriginal
- ◯ Mixed race
- ◯ Unknown
- ◯ Other (please specify)

The STARS Study

When was this baby born?

Please enter date MM [ ] / DD [ ] / YYYY [ ]

What is this baby's gender?

- ◯ Male
- ◯ Female
- ◯ Uncertain

Please answer only one of the following three questions depending on whether you prefer to answer in pounds & ounces, kilos or grams.

POUNDS/OUNCES - What was your baby's weight in pounds and ounces?

	Pounds	Ounces
Please enter baby's birth weight in pounds & ounces.		

KILOS - What was your baby's birth weight in kilos?

	Kilos
Baby's birth weight in kilos.	

GRAMS - What was your baby's birth weight in grams?

The STARS Study

Where did you deliver this baby?

- Hospital
- Out of hospital birth center
- At Home

Other (please specify)

How many weeks pregnant were you when this baby was born?

- 28
- 29
- 30
- 31
- 32
- 33
- 34
- 35
- 36
- 37
- 38
- 39
- 40
- 41
- 42 or more
- Uncertain, please explailn

Which of the following describes your main healthcare provider during this pregnancy?

- Obstetrician
- Family Physician
- Midwife - CNM (United States)
- Midwife - CPM (United States)
- Midwife (International Provider)
- Maternal/Fetal Medicine Specialist
- Perinatologist
- Did not have prenatal care during this pregnancy

Other (please specify)

Did you change healthcare providers at any time during this pregnancy?

- No
- Yes, (please explain)

What was your weight before this pregnancy? (Please specify either pounds/ounces or kilos)

	Pounds	Kilos
Enter closest pre-pregnancy weight		

The STARS Study

How much weight did you gain during this pregnancy? (Please specify pounds/ounces or kilograms)

	Pounds	Ounces	Kilos
Enter weight gained during this pregnancy.			

The STARS Study

How many weeks pregnant were you when you first visited a healthcare provider about your pregnancy?

	Weeks
Select Number of Weeks Pregnant	

Were you considered 'high risk' for this pregnancy?

- ◯ No
- ◯ Yes, due to a medical condition diagnosed before pregnancy
- ◯ Yes, due to a medical condition diagnosed during pregnancy
- ◯ Yes, due to a previous pregnancy complication
- ◯ Yes, due to a previous pregnancy loss
- ◯ I don't know

If yes, please describe

Did you have any medical conditions prior to this pregnancy?

- ◯ No
- ◯ Yes

If yes, please describe

Were you diagnosed with or treated for any medical conditions during this pregnancy?

- ◯ No
- ◯ Yes

If yes, please describe

The STARS Study

Did you use fertility treatments to become pregnant with this baby?

- ◯ No
- ◯ Yes

If yes, please describe:

The STARS Study

How many pregnancies did you have BEFORE this pregnancy?

- ◯ 0
- ◯ 1
- ◯ 2
- ◯ 3
- ◯ 4
- ◯ 5
- ◯ 6
- ◯ 7
- ◯ 8
- ◯ 9
- ◯ 10
- ◯ More than 10

How many live children have you given birth to BEFORE this pregnancy?

- ◯ 0
- ◯ 1
- ◯ 2
- ◯ 3
- ◯ 4
- ◯ 5
- ◯ 6
- ◯ 7
- ◯ 8
- ◯ 9
- ◯ 10
- ◯ More than 10

Before this pregnancy did you have any pregnancies end:

	Before 12 weeks	12-27 weeks	28 or more weeks
Please indicate how many previous losses you have had for the time period indicated.			

Did this baby have a congenital abnormality (genetic condition, physical abnormality, etc.)?

- ◯ Yes
- ◯ No
- ◯ I don't know

If yes, please describe

Was this pregnancy ever a multiple gestation pregnancy (more than one baby)?

- ◯ No
- ◯ Yes - twins (2)
- ◯ Yes - triplets (3)
- ◯ Yes - quadruplets (4)
- ◯ Yes - more than 4 babies

If yes to the previous question, were any of the babies from this pregnancy born alive?

- ◯ No
- ◯ Yes - 1 baby survived
- ◯ Yes - 2 babies survived
- ◯ Yes - 3 babies survived
- ◯ Yes - 4 babies survived
- ◯ Yes - more than 4 babies survived
- ◯ Not applicable

How would you describe your activity level BEFORE this pregnancy?

◯ INACTIVE - rarely walk anywhere, have a sedentary job that involves sitting for long periods, and generally you don't do any specific exercise during an average day.

◯ LIGHT ACTIVITY - You might have a light walk to and from work, or you may have a job that means you spend periods of time on your feet or walking around. You may also occasionally visit a gym or take part in exercise classes.

◯ MODERATE ACTIVITY - you exercise regularly for 30 minutes or more, up to 5 x/week, you will also probably have a hobby that involves moderate activity, or maybe you take a dog for a long walk several times a week .

◯ ACTIVE - regular exercise for 30 minutes or more, 5 x/week or more. You are also likely to walk often during your day and have a hobby that includes involves a high level of activity.

How would you describe your usual activity level DURING this pregnancy?

◯ INACTIVE - rarely walk anywhere, have a sedentary job that involves sitting for long periods, and generally you don't do any specific exercise during an average day.

◯ LIGHT ACTIVITY - You might have a light walk to and from work, or you may have a job that means you spend periods of time on your feet or walking around. You may also occasionally visit a gym or take part in exercise classes.

◯ MODERATE ACTIVITY - you exercise regularly for 30 minutes or more, up to 5 x/week, you will also probably have a hobby that involves moderate activity, or maybe you take a dog for a long walk several times a week .

◯ ACTIVE - regular exercise for 30 minutes or more, 5 x/week or more. You are also likely to walk often during your day and have a hobby that includes involves a high level of activity.

◯ MEDICALLY PRESCRIBED BED REST - due to a concern with your pregnancy you were told to remain inactive on bedrest for a significant time during this pregnancy.

Did you have any ultrasound examinations during this pregnancy?

- ◯ No
- ◯ Yes, one.
- ◯ Yes, two.
- ◯ Yes, three or more.

If you answered yes to the previous question, were any of these ultrasounds a Level 2 (anatomy examination) ultrasound or done with a biophysical profile (a longer ultrasound watching for baby's breathing-like activity and movements).

- ◯ Yes
- ◯ No
- ◯ I don't know.
- ◯ Not applicable.

During an ultrasound, were any abnormalities or concerns identified?

- ◯ No
- ◯ Yes
- ◯ I do not know
- ◯ Not applicable

If yes, please describe

What was the position of the placenta noted during ultrasound?

- ◯ Anterior
- ◯ Posterior
- ◯ Fundal
- ◯ Lateral
- ◯ Previa
- ◯ Other
- ◯ I don't know

Please describe:

During an ultrasound examination, did your healthcare provider comment on any other characteristics of your baby's umbilical cord before birth? (Choose all that apply)

- ☐ I don't remember
- ☐ No
- ☐ Yes, the cord was around the baby's neck
- ☐ Yes, there was extra twisting of the cord
- ☐ Yes, there was no twisting of the cord
- ☐ Yes, abnormal insertion into the placenta
- ☐ Yes, cord was wrapped around body parts other than the neck
- ☐ Yes, a knot(s) was identified in the cord
- ☐ Yes, the umbilical cord only had two blood vessels

Other (please specify)

____

Did you experience any vaginal bleeding during this pregnancy?

- ○ No
- ○ Yes, a single episode before 20 weeks.
- ○ Yes, recurrent bleeding before 20 weeks.
- ○ Yes, single episode after 20 weeks.
- ○ Yes, recurrent bleeding after 20 weeks.
- ○ Yes, recurrent bleeding throughout the pregnancy.
- ○ I don't remember.

Do you smoke tobacco?

- ○ Yes
- ○ No, I have never smoked.
- ○ No, I stopped before this pregnancy.
- ○ No, I stopped during pregnancy.
- ○ I tried to quit but restarted during pregnancy.
- ○ I quit smoking but used nicotine patches.

If you smoked at any time during this pregnancy, how many cigarettes did you smoke on average each day?

____

Does anyone living in your household (other than yourself) smoke?

- ○ No
- ○ Yes

The STARS Study

During this pregnancy did you work in an environment where you were exposed to second hand smoke?

- ◯ Yes
- ◯ No

During this pregnancy, did you use any recreational drugs (marijuana, cocaine, etc)?

- ◯ No
- ◯ Yes, once
- ◯ Yes, occasionally
- ◯ Yes, frequently

During this pregnancy, did you use any of the following:

	Never	1-2 times throughout pregnancy	monthly	weekly	daily
Alcohol	◯	◯	◯	◯	◯
Prescripition drugs	◯	◯	◯	◯	◯
Over the counter drugs	◯	◯	◯	◯	◯
Vitamins	◯	◯	◯	◯	◯
Dietary supplements	◯	◯	◯	◯	◯
Herbal remedies	◯	◯	◯	◯	◯
Folk remedies	◯	◯	◯	◯	◯
Complimentary therapies	◯	◯	◯	◯	◯
Alternative therapies	◯	◯	◯	◯	◯
Naturopathic therapies	◯	◯	◯	◯	◯
Holistic therapies	◯	◯	◯	◯	◯

Please describe your answers (other than never)

The STARS Study

What range is your usual blood pressure when you are not pregnant?

- ◯ Very low
- ◯ Borderline low
- ◯ Normal
- ◯ Borderline high
- ◯ Very high
- ◯ I don't know

If you know your usual blood pressure when not pregnant please provide it here

[ ]

The STARS Study

During this pregnancy, in what range was your blood pressure?

- ◯ Low
- ◯ Borderline low
- ◯ Normal
- ◯ Borderline high
- ◯ High

If you know your usual blood pressure during this pregnancy please provide it here

[ ]

The STARS Study

In the next several questions, "last night of the pregnancy" refers to the last night before you this your baby died or were told your baby had died.

On average, what time did you usually go to bed BEFORE this pregnancy?

	Hour	AM or PM
Usual bedtime before pregnancy		

On average, what time did you get up each day BEFORE this pregnancy?

	Hour	AM or PM
Indicate the time you got up BEFORE this pregnancy.		

On average, how many hours of sleep did you get BEFORE this pregnancy (this may be different than the number of hours spent in bed)?

	Hours
Hours of sleep before pregnancy	

On average, how many times would you WAKE up during the night?

	None	1 x/night	2 x/night	3-4 x/night	5 or more x/night	I don't remember
Before this pregnancy	○	○	○	○	○	○
Last month of this pregnancy	○	○	○	○	○	○
Last night of this pregnancy	○	○	○	○	○	○

On average, how many times would you GET UP during the night?

	None	1 x/night	2 x/night	3-4 x/night	5 or more x/night	I don't remember
Before this pregnancy	○	○	○	○	○	○
Last month of this pregnancy	○	○	○	○	○	○
Last night of this pregnancy	○	○	○	○	○	○

On average, what time did you go to bed the LAST MONTH of this pregnancy?

	Hour	AM or PM
Bedtime last month of this pregnancy		

On average, what time did you get up in the morning in the LAST MONTH of this pregnancy?

	Hour	AM or PM
Time you got up in last month of pregnancy		

On average, how many hours of sleep did you get at night during this pregnancy?

	Hours
Hours of sleep during this pregnancy	

On the LAST NIGHT of this pregnancy, what time did you go to bed?

	Hour	AM or PM
Bedtime on last night of this pregnancy		

The STARS Study

On the LAST NIGHT of this pregnancy, what time did you get up in the morning?

	Hour	AM or PM
Time you got up on last night of this pregnancy		

On the LAST NIGHT of this pregnancy, how many hours of sleep did you get (may not be the same as the number of hours spent in bed)?

	Hours
Hours of sleep last night of this pregnancy	

On the last night of this pregnancy, did your partner sleep in the same bed with you?

- ◯ Yes
- ◯ No
- ◯ No partner
- ◯ I don't remember.

On the last night of this pregnancy, which side of the bed did you sleep on?

- ◯ Left
- ◯ Right
- ◯ Middle
- ◯ I don't remember

In what position did you fall asleep?

	Left	Back	Right	Tummy/Stomach	Variable	Propped	I don't remember.
Before this pregnancy	◯	◯	◯	◯	◯	◯	◯
Last month of this pregnancy	◯	◯	◯	◯	◯	◯	◯
Last night of this pregnancy	◯	◯	◯	◯	◯	◯	◯

In what position did you wake up?

	Left	Back	Right	Tummy/Stomach	Variable	Propped	I don't remember.
Before this pregnancy	◯	◯	◯	◯	◯	◯	◯
Last month of this pregnancy	◯	◯	◯	◯	◯	◯	◯
Last night of this pregnancy	◯	◯	◯	◯	◯	◯	◯

Did you change positions during the night?

	Not at all	Possibly once	Possibly twice	More than twice but not lots	Lots of times	I don't remember
Before this pregnancy	◯	◯	◯	◯	◯	◯
Last month of this pregnancy	◯	◯	◯	◯	◯	◯
Last night of this pregnancy	◯	◯	◯	◯	◯	◯

The STARS Study

Would you describe yourself as a restless sleeper (move a lot during the night)?

	Not at all	A little	Average	More than average	Very restless
Before this pregnancy	○	○	○	○	○
Last month of this pregnancy	○	○	○	○	○
Last night of this pregnancy	○	○	○	○	○

BEFORE this pregnancy did you snore or were you ever told that you snored?

○ Yes

○ No

DURING this pregnancy, did you snore or were you told that you snored?

○ Yes

○ No

How often did you snore or were you told that you snore?

	Rarely	Sometimes	Often	Every night	I don't know	Didn't snore
Before this pregnancy	○	○	○	○	○	○
Last month of this pregnancy	○	○	○	○	○	○
Last night of this pregnancy	○	○	○	○	○	○

Were you told your snoring ever bothered other people?

	Yes	No
Before this pregnancy	○	○
Last month of this pregnancy	○	○
Last night of this pregnancy	○	○

How loud was your snoring?

	Slightly louder than breathing	As loud as talking	Louder than talking	Very loud, could be heard in adjacent rooms	I don't know	Didn't snore
Before this pregnancy	○	○	○	○	○	○
Last month of this pregnancy	○	○	○	○	○	○
Last night of this pregnancy	○	○	○	○	○	○

Were you ever told that you stopped breathing or gasped for air during sleep?

	Never	Rarely	Sometimes	Often	Every night
Before this pregnancy	○	○	○	○	○
Last month of this pregnancy	○	○	○	○	○

Were you told you stopped breathing or gasped for air during the last night of this pregnancy?

- ◯ Yes
- ◯ No

Were you ever told that you cough or choke during sleep?

	Never	Rarely	Sometimes	Often	Every night
Before this pregnancy	◯	◯	◯	◯	◯
Last month of this pregnancy	◯	◯	◯	◯	◯

Were you told you cough or choked during the last night of this pregnancy?

- ◯ Yes
- ◯ No

Did your legs twitch or jerk often while you slept?

	Never	Rarely	Sometimes	Often	Every night	I don't remember
Before this pregnancy	◯	◯	◯	◯	◯	◯
Last month of this pregnancy	◯	◯	◯	◯	◯	◯
Last night of this pregnancy	◯	◯	◯	◯	◯	◯

Did your legs twitch or jerk often during the last night of this pregnancy?

- ◯ Yes
- ◯ No
- ◯ I don't remember

Did you have unpleasant sensations in your legs combined with the need to move your legs?

	Yes	No	I don't remember
Before this pregnancy	◯	◯	◯
Last month of this pregnancy	◯	◯	◯
Last night of this pregnancy	◯	◯	◯

If yes to the last question, do these feelings occur mainly or only at rest and do they improve with movement?

	Yes	No	I don't remember	Not applicable
Before this pregnancy	◯	◯	◯	◯
Last month of this pregnancy	◯	◯	◯	◯
Last night of this pregnancy	◯	◯	◯	◯

Are these feelings worse in the evening/night than the morning?

	Yes	No	I don't remember	Not applicable
Before this pregnancy	○	○	○	○
Last month of this pregnancy	○	○	○	○
Last night of this pregnancy	○	○	○	○

How often do these feelings occur?

	I don't remember	Never	<1x/year	At least 1x/yr but <1x/mo	1x/mo	2-4x/mo	2-3x/wk	4-5x/wk	6-7x/wk
Before this pregnancy	○	○	○	○	○	○	○	○	○
Last month of this pregnancy	○	○	○	○	○	○	○	○	○

Did you take medication/herbal remedies for sleep (either prescription or over the counter)?

	Never	Occasionally	1-2 x/week	3-4 x/week	Every night
Before this pregnancy	○	○	○	○	○
Last month of this pregnancy	○	○	○	○	○

Did you take medication/herbal remedies for sleep on the last night of this pregnancy (either prescription or over the counter)?

○ Yes

○ No

If yes, please specify

[ ]

How would you rate your overall sleep quality?

	Very good	Fairly good	Average	Faily bad	Very bad
Before this pregnancy	○	○	○	○	○
Last month of this pregnancy	○	○	○	○	○
Last night of this pregnancy	○	○	○	○	○

How often did you feel fatigued or tired after your nights sleep?

	Never	Rarely	Sometimes	Often	Every night	I don't know
Before this pregnancy	○	○	○	○	○	○
Last month of this pregnancy	○	○	○	○	○	○

During your waking hours, did you feel tired or fatigued?

	Never	Rarely	Sometimes	Often	Every day	I don't know
Before this pregnancy	○	○	○	○	○	○
Last month of this pregnancy	○	○	○	○	○	○

The STARS Study

Before this pregnancy, how likely were you to doze off or fall asleep in the following situations (as opposed to just feeling tired)?

	Never	Slight chance	Moderate chance	High chance
Sitting and reading	○	○	○	○
Watching TV	○	○	○	○
Sitting inactive in a public place	○	○	○	○
Passenger in a car for one hour without a break	○	○	○	○
Lying or sitting down to rest in the afternoon	○	○	○	○
Sitting and talking to someone	○	○	○	○
Sitting quietly after lunch without alcohol	○	○	○	○
In a car while stopped for a few minutes in traffic	○	○	○	○

In the last month of this pregnancy, how likely were you to doze off or fall asleep in the following situations?

	Never	Slight chance	Moderate chance	High chance
Sitting and reading	○	○	○	○
Watching TV	○	○	○	○
Sitting inactive in a public place	○	○	○	○
Passenger in a car for an hour without a break	○	○	○	○
Lying or sitting down to rest in the afternoon	○	○	○	○
Sitting and talking to someone	○	○	○	○
Sitting quietly after lunch without alcohol	○	○	○	○
In a car while stopped for a few minutes in traffic	○	○	○	○

On average how many times would you take a nap during the day?

	Never	Rarely	Occasionally	Often	Every day	I don't know.
Before this pregnancy	○	○	○	○	○	○
Last month of this pregnancy	○	○	○	○	○	○

On average how long would your nap be before this pregnancy? Select 0 if you did not nap.

	Hours
Length of naps before pregnancy	

On average how long would your nap be the last month of this pregnancy? Select 0 if you did not nap during the last month of this pregnancy.

	Hours
Length of naps in last month of this pregnancy	

The next questions ask about your baby's movements.

During this pregnancy did your healthcare provider tell you about or ask you to keep track of your baby's movement.

- ◯ Yes
- ◯ No
- ◯ I don't remember

Did you keep track of your baby's movement during this pregnancy?

- ◯ Yes
- ◯ No

If yes, please describe the method you used to keep track of your baby's movements

How would you describe this baby's usual movements?

- ◯ Little to no movement felt
- ◯ Infrequent movements
- ◯ Average movements
- ◯ Above average movements
- ◯ Constant movement
- ◯ I don't remember

Once you were aware of your baby's usual pattern of movement, was there any time your baby's movements were unusual?

- ◯ No
- ◯ Yes, a little bit more
- ◯ Yes, significantly more
- ◯ Yes, a little bit less
- ◯ Yes, significantly less
- ◯ I don't remember

If yes, please explain

If you answered yes to the previous question, which of the following describes your experience?

- ◯ Did not worry about it
- ◯ Mentioned to family/friends but did not worry
- ◯ Mentioned to my healthcare provider and was reassured
- ◯ Mentioned to my healthcare provider and was told to monitor for symptoms at home or call if more concerned
- ◯ Mentioned to my healthcare provider and was evaluated with a general examination (baby's heart rate, amount of cervical dialation/effacement, etc)
- ◯ Mentioned to my healthcare provider and was evaluated with ultrasound, non-stress test, biophysical profile or similar testing
- ◯ Mentioned to my health care provider and was admitted to emergency room and/or hospital for testing and/or monitoring
- ◯ Went to the emergency room or labor & delivery and was sent home after evaluation
- ◯ Went to the emergency room or labor & delivery and was admitted for testing and/or monitorinig
- ◯ Not applicable

Other/comments

[ ]

During the last two weeks of this pregnancy, were the STRENGTH of your baby's movements....

- ◯ Increased
- ◯ Decreased
- ◯ Stayed the same
- ◯ I don't remember

Please describe:

[ ]

During the last two weeks of this pregnancy, did the FREQUENCY of your baby's movements....

- ◯ Increase
- ◯ Decrease
- ◯ Stay the same
- ◯ I don't remember

Please describe:

[ ]

Did you usually feel your baby move at bedtime during this pregnancy?

- Yes
- No
- I don't remember

Did you feel your baby move at bedtime on the last night of this pregnancy?

- Yes
- No
- I don't remember

During the last two weeks of this pregnancy, did you notice any time that your baby was more vigorous than usual?

- No
- Yes, once.
- Yes, sometimes.
- Yes, often.
- I don't remember.

If yes, please describe:

Did you experience your baby having hiccup like movements?

- yes
- no
- I don't remember

The STARS Study

If you noticed your baby having hiccup like movements, how long would each episode last on average?

- ◯ Less than 5 minutes
- ◯ 5-10 minutes
- ◯ More than 10 minutes
- ◯ I don't remember

If your baby experienced hiccup like movements, how often did you notice them?

- ◯ Once or twice thought this pregnancy
- ◯ Weekly
- ◯ Daily
- ◯ More than 3 times per day
- ◯ I don't remember

Please describe:

If you experienced a change in your baby's behavior, which of the following best describes your experience (check all that apply)

- ☐ Did not worry about it
- ☐ Mentioned to family/friends but didn't not worry
- ☐ Mentioned to my healthcare provider and was reassured
- ☐ Mentioned to my healthcare provider and was told to monitor for symptoms at home or call again if more concerned
- ☐ Mentioned to my healthcare provider and was evaluated by checking the baby's heart beat
- ☐ Mentioned to my healthcare proficer and was evaluated with ultrasound, non-stress test, biophysical profile or similar testing.
- ☐ Mentioned to my healthcare provider and was admitted to emergency room or hospital for testing and monitoring
- ☐ Went to emergency room or labor and delivery and was sent home.
- ☐ Went to emergency room or labor and delivery and was evaluated with ultrasound and/or fetal heart rate monitoring.
- ☐ Not applicable.

During the last two weeks of this pregnancy, did you feel contractions, pre-labor contractions, Braxton-Hick contractions or false labor for longer than an hour at any one time?

- ◯ yes
- ◯ No

If you answered yes to the previous question, which of the following describes your experience?

- ◯ Did not worry about it
- ◯ Mentioned to family/friends but did not worry
- ◯ Mentioned to my healthcare provider and was reassured
- ◯ Mentioned to my healthcare provider and was told to monitor for symptoms at home or call if more concerned
- ◯ Mentioned to my healthcare provider and was evaluated with a general examination (baby's heart rate, amount of cervical dialation/effacement, etc)
- ◯ Mentioned to my healthcare provider and was evaluated with ultrasound, non-stress test, biophysical profile or similar testing
- ◯ Mentioned to my health care provider and was admitted to emergency room and/or hospital for testing and/or monitoring
- ◯ Went to the emergency room or labor & delivery and was sent home after evaluation
- ◯ Went to the emergency room or labor & delivery and was admitted for testing and/or monitorinig
- ◯ Not applicable

Other/comment:

[ ]

During this pregnancy did you ever have a "gut instinct" that something was not right?

- ◯ yes
- ◯ no

If yes, please describe

[ ]

Were you TESTED for any viral or bacterial infections during this pregnancy?

- Yes
- No
- I don't know.

If yes, please describe

Were you TREATED for any infections during this pregnancy?

- No
- Yes
- I don't know

If yes, please describe

If you were treated for an infection, were you retested to be sure the infection was gone?

- Yes
- No
- I don't know
- Not applicable

Were your membranes stripped or swept by your healthcare provider during this pregnancy?

- No
- I don't know
- Yes

If yes, how long was it done before the baby was born?

If your membranes were stripped or swept, please tell us why this was done.

The STARS Study

Did you take any antibiotics during this pregnancy?

- ◯ No
- ◯ Yes, during the first trimester
- ◯ Yes, during the second trimester
- ◯ Yes, during the third trimester
- ◯ Yes, during labor
- ◯ I don't know

If yes, what were the antibiotics given to you for?

If yes to the previous question, what was the name of the antibiotic you were given. Leave blank if you do not remember.

Were you sexually active during this pregnancy?

- ◯ No
- ◯ Yes, rarely
- ◯ Yes, occasionally
- ◯ Yes, frequently

Did you have more than one sexual partner during this pregnancy?

- ◯ Yes
- ◯ No

Was an autopsy or other post mortem examination performed? (Select all that apply.)

- ☐ No
- ☐ Yes, placenta
- ☐ Yes, umbilical cord
- ☐ Yes, non-invasive exam of baby
- ☐ Yes, blood tests on baby
- ☐ Yes, blood tests on Mom
- ☐ Yes, chromosome/genetic studies
- ☐ Yes, tissue samples
- ☐ Yes, full autopsy
- ☐ Yes, imaging (xray, MRI, CT, etc.)
- ☐ Other: (please describe)

Did your healthcare provider comment on the length of your baby's umbilical cord?

- ○ No
- ○ Yes, it was shorter than normal.
- ○ Yes, it was normal length
- ○ Yes, was longer than normal

Did your healthcare provider comment on any other characteristics of your baby's umbilical cord after delivery?

- ◯ I don't remember
- ◯ No
- ◯ Yes, the cord was around my baby's neck
- ◯ Yes, there was extra twisting of the cord
- ◯ Yes, there was no twisting of the cord
- ◯ Yes, the cord was abnormally inserted into the placenta
- ◯ Yes, the cord was wrapped around body parts other than the neck
- ◯ Yes, there was a knot(s) in the cord
- ◯ Yes, there was a combination of two or more of the above issues

If yes please describe

What were you told caused your baby's death?

[ ]

If you had an ultrasound during this pregnancy, how many weeks pregnant were you when the last routine ultrasound was done before this baby died? (Do not answer for an ultrasound that was done to confirm your baby's death but rather the one most previous to your baby's death.)

[ ]

What do you believe caused your baby's death?

[ ]

When do you believe this baby died? (Leave blank if you do not know)

Date MM / DD / YYYY

When did you first know that your baby was in trouble or had died?

- [ ] I felt a reduction of kicks/movements.
- [ ] I felt kicks/movements stop.
- [ ] I had abdominal pain.
- [ ] I had vaginal bleeding.
- [ ] I had discharge of amniotic fluid/membranes ruptured/water broke.
- [ ] I had a "feeling that something was wrong" but couldn't specify.
- [ ] I had other symptoms - please specify below.
- [ ] I had physical trauma.
- [ ] I was told at an appointment for prenatal care.
- [ ] I was told when admitted for labor.
- [ ] I was told during labor.
- [ ] It was not discovered before my baby was born.
- [ ] I do not remember.

Other - please specify other symptoms

[ ]

The STARS Study

What time do you believe your baby died?

- ◯ In the morning (6am - 12 noon)
- ◯ In the afternoon (12 noon - 6pm)
- ◯ In the evening (6pm - 10 pm)
- ◯ During the night (10pm - 6am)
- ◯ During a daytime nap
- ◯ I am not sure.
